# Digitoxin in heart failure: a statistical signal, or just noise? A reappraisal of the DIGIT-HF trial

**DOI:** 10.1093/ehjcvp/pvaf089

**Published:** 2025-12-16

**Authors:** Ahmed Abdelaziz, Ibrahim Halil Tanboga, Gregg C Fonarow

**Affiliations:** Division of Cardiology, Montefiore Health System/Albert Einstein College of Medicine, Bronx, NY 10467, USA; Department of Cardiology, Hisar Intercontinental Hospital, Istanbul 34768, Turkey; Department of Biostatistics and Cardiology, Nisantasi University Medical School, Istanbul 34768, Turkey; Ahmanson-UCLA Cardiomyopathy Center, Ronald Reagan-UCLA Medical Center, Los Angeles, CA 90095, USA

## Abstract

**Graphical Abstract:**
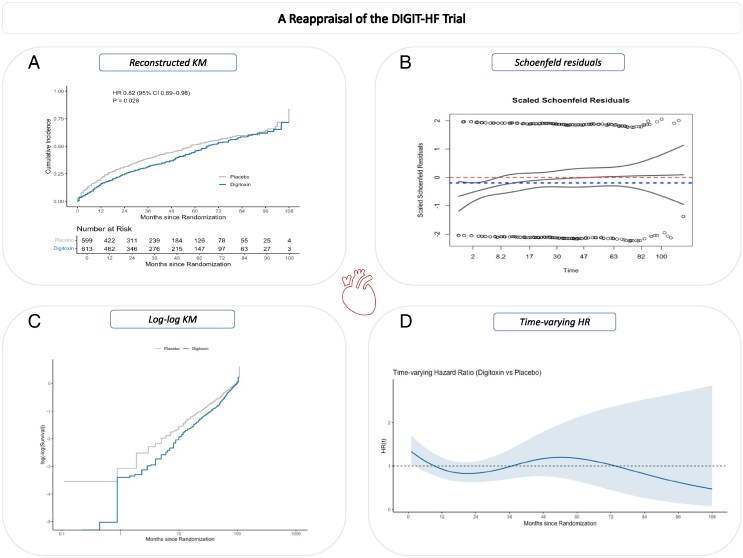
Reconstructed time-to-event analyses from the published DIGIT-HF Kaplan–Meier curves, demonstrating early clinical benefits with digitoxin and minor late phase non-proportionality consistent across methods. (*A*) Reconstructed Kaplan–Meier curve showing early divergence and late convergence, (*B*) Schoenfeld residuals plot demonstrating minor non-proportionality, (*C*) Log–log survival plot confirming overall parallelism between groups, and (*D*) Time-varying hazard ratio estimated with restricted cubic splines (3 knots).

Reconstructed time-to-event analyses from the published DIGIT-HF Kaplan–Meier curves, demonstrating early clinical benefits with digitoxin and minor late phase non-proportionality consistent across methods. (*A*) Reconstructed Kaplan–Meier curve showing early divergence and late convergence, (*B*) Schoenfeld residuals plot demonstrating minor non-proportionality, (*C*) Log–log survival plot confirming overall parallelism between groups, and (*D*) Time-varying hazard ratio estimated with restricted cubic splines (3 knots).

Although the Digitalis Investigation Group (DIG) trial,^1^ showed that digoxin significantly reduced hospitalization rates for worsening heart failure (HF), but not mortality, in patients with HF and reduced ejection fraction (HFrEF), other treatments such as beta-blockers, mineralocorticoid receptor antagonists (MRAs), and sodium-glucose co-transporter 2 (SGLT2) inhibitors improved both outcomes. Consequently, these treatments received strong guideline recommendations, and the use of digoxin, relegated to second-line therapy, has declined.^2^ The DIGIT-HF trial recently re-examined the role of cardiac glycosides, added to these other therapies, using the alternative agent digitoxin.^3^ The DIGIT-HF investigators reported a significant reduction in the primary composite endpoint of all-cause mortality or hospitalization for worsening heart failure (HR: 0.82; 95% CI: 0.69–0.98; *P* = 0.03). The published Kaplan–Meier (KM) displayed late convergence, which may raise questions related to non-proportional hazards assumptions. While this does not imply an analytical error, we thought of revisiting the statistical considerations relevant to long-term survival analysis.

## Kaplan–Meier curves: divergence, convergence, and proportional hazards

The survival analysis of KM curves reported in the DIGIT-HF trial revealed an early phase of divergence favoring digitoxin until 48 months of follow-up, subsequent by a phase of attenuation and eventual convergence. To test the non-proportionality (NP), we first reconstructed the individual-patient data (IPD) from the published KM curves of the DIGIT-HF trial using WebPlotDigitizer (v5.2) and validated against the published reported event counts and hazard ratios (HRs), to ensure consistency. Detailed methodologies with the required codes are provided in the Supplementary material online, *Appendix*. Subsequently, visual assessments and *P*-value-based evaluations were performed.^4^ Grambsch and Thernau’s test for NP based on *P*-value revealed a *P*-value for NP of 0.025, indicating there was NP. However, such minor deviations are common in clinical trials with extended follow-up and don’t materially alter the effect estimate. In addition, visual assessments as log–log KM survival estimates and Schoenfeld residuals indicated that the NP was minor (*Graphical Abstract*). The methodology on the reconstruction procedure and reproducibility materials is found in the Supplementary material online, *Appendix*.

## Supplementary evidence to cox proportional hazard model

In further exploratory analyses, we tested HR over the study follow-up durations, and the HR was relatively constant, despite ignoring the small fluctuations, which were accounted for in the time model using restricted cubic splines with three knots (*Graphical Abstract*). Additionally, to assess the time dependency, we estimated restricted mean survival time (RMST) difference at 36 months was 2.20 months (95% CI 0.76–3.63, *P* = 0.003), and 4.44 months (95% CI 1.10–7.77, *P* = 0.009) at 72 months, highlighting that, on average, the expected time until patients in the digitoxin group experienced the event was 2.2 months longer than those in the placebo group at 36 months, and 4.44 months longer at 72 months.

In addition, to complement the framework of proportional hazards, we explored accelerated failure time (AFT) model, the placebo group showed shorter survival compared with digitoxin (Time Ratio = 1.29, 95% CI: 1.03–1.61, *P* = 0.023), indicating ∼22% shorter event-free time. Similarly, parametric AFT methods, assuming log-logistic and log-normal distributions, yielded consistent results: log-normal AFT (Time ratio = 1.57, 95% CI 1.20–2.07, *P* = 0.0012) and log-logistic AFT (Time ratio = 1.40, 95% CI 1.09–1.79, *P* = 0.0076). Across all models, the magnitude and direction of the digitoxin effect remained consistent, supporting the robustness of the original findings of the DIGIT-HF trial. These AFT assumptions should be interpreted as complementary rather than superiority of the Cox proportional model, supporting the original findings of the trial conclusion.

## Attrition and the fragility of long-term estimates

In the DIGIT-HF trial, by 60 months of follow-up, fewer than 150 patients remained at risk, and by 72 months, interpretability was limited due to attrition and censoring. As evident by Pocock *et al*.,^5^ such late phase curve reflects sparsity of data rather than true loos of efficacy, of which this highlights the need for interpreting the present long-term survival data in terms of risk attrition rather than a simple change in the digitoxin effect. Recent HFrEF trials, including PARADIGM-HF, DAPA-HF, and EMPEROR-Reduced^6-8^ truncated their follow-up durations around 24–36 months when the number at risk drops below 10%, avoiding the misleading data in late tails.

## Conclusion

We conclude that the DIGIT-HF trial remains a positive and statistically robust trial. While minor deviations from proportional hazards were identified, they are common in long-term trials with survival analysis and have no significant effect on the primary interpretations. Our supplementary methods aim to provide clinicians with a practical framework for interpreting the survival analysis data in extended follow-up studies. Additionally, a Bayesian approach in the light of DIG might also be interesting to explore.

## Supplementary Material

pvaf089_Supplementary_Data

## Data Availability

The data underlying this article are available in the article.
