# Supplementary material for: Digitoxin in heart failure: a statistical signal, or just noise? A reappraisal of the DIGIT-HF trial
Source: Eur Heart J Cardiovasc Pharmacother. 2025 Dec 16;12(2):85–7. doi: 10.1093/ehjcvp/pvaf089 (PMC12946962; doi:10.1093/ehjcvp/pvaf089)
Supplement: pvaf089_Supplementary_Data [file pvaf089_supplementary_data.doc]

**Supplementary Appendix**

**First Step: Data Extraction and Reconstruction**

Kaplan–Meier Curve Digitization: Kaplan–Meier curves for the primary outcome were extracted from the published DIGIT-HF paper using WebPlotDigitizer (version 5.2). Curves were carefully digitized to obtain time-to-event coordinates for all reported time points.

Individual Patient Data (IPD) Reconstruction: Individual patient data were reconstructed from the digitized coordinates using the algorithm described by Guyot et al. [1]. The reconstruction process included the following steps:

1. Extraction of time–event coordinates for all KM curves.
2. Calculation of the number of patients at risk and event counts at each time interval.
3. Reconstruction of individual survival times based on the extracted cumulative survival probabilities.
4. Formatting reconstructed IPD into a dataset suitable for survival analyses.

Validation of Reconstructed Data: The reconstructed dataset was cross-checked against the original publication for:

- Number of events reported at each time interval
- Hazard ratios and confidence intervals for the primary outcome

Minor discrepancies were within acceptable limits for graphical reconstruction (<3%), confirming the reliability of the reconstructed IPD.

**Second Step: Statistical Analyses**

Survival Analyses: All analyses were performed in R (version X.X). The following approaches were applied to the reconstructed IPD:

Cox Proportional Hazards (PH) Models: Hazard ratios for the primary outcome were calculated, and proportional hazards' assumptions were tested using Schoenfeld residuals.

Restricted Mean Survival Time (RMST): RMST differences were estimated for pre-specified time horizons to complement Cox PH analyses.

Accelerated Failure Time (AFT) Models: AFT models were applied to assess potential non-proportionality and provide alternative survival estimates.

Non-Proportionality Checks: Graphical inspection and statistical tests (Schoenfeld residuals) were used to evaluate the proportional hazards assumption for all Cox models.

**Third Step: R Script for IPD Reconstruction and Survival Analyses**

The following R script reproduces all analyses reported in the manuscript, including IPD reconstruction, Cox proportional hazards models, non-proportionality checks, RMST, AFT models, and Kaplan–Meier curves:

# **Load required libraries**

library(survival) # Cox PH and AFT models library(survminer) # Kaplan-Meier plots library(rmst2) # Restricted Mean Survival Time analyses library(dplyr) # Data manipulation library(readr) # CSV import/export library(ggplot2) # Custom plotting

# **1. Load reconstructed IPD**

# **The dataset should contain: id, time, event (0=censor, 1=event), treatment**

ipd <- read_csv("digitized_ipd.csv")

# **Inspect dataset**

head(ipd) summary(ipd)

# **2. Cox Proportional Hazards Model**

cox_model <- coxph(Surv(time, event) ~ treatment, data = ipd) summary(cox_model)

# **Check proportional hazards assumption**

ph_test <- cox.zph(cox_model) ph_test plot(ph_test) # Visual inspection of proportional hazards

# **3. Kaplan-Meier Curves**

km_fit <- survfit(Surv(time, event) ~ treatment, data = ipd)

# **Basic KM plot with risk table and confidence intervals**

ggsurvplot( km_fit, data = ipd, risk.table = TRUE, pval = TRUE, conf.int = TRUE, palette = c("#E7B800", "#2E9FDF") )

# **4. Restricted Mean Survival Time (RMST)**

# **Set truncation time (e.g., max follow-up)**

tau <- max(ipd$time)

rmst_result <- rmst2(time = ipd$time, status = ipd$event, arm = ipd$treatment, tau = tau) print(rmst_result)

# **5. Accelerated Failure Time (AFT) Models**

# **Weibull AFT model**

aft_model <- survreg(Surv(time, event) ~ treatment, data = ipd, dist = "weibull") summary(aft_model)

# **Optional log-normal AFT model**

aft_model_ln <- survreg(Surv(time, event) ~ treatment, data = ipd, dist = "lognormal") summary(aft_model_ln)

# **6. Survival Curves from Cox Model**

newdata <- data.frame(treatment = unique(ipd$treatment)) surv_fit <- survfit(cox_model, newdata = newdata)

ggsurvplot( surv_fit, data = newdata, conf.int = TRUE, legend.labs = c("Treatment", "Control"), palette = c("#E7B800", "#2E9FDF"), xlab = "Time (days)", ylab = "Survival Probability" )

# **7. Save Figures and Results**

ggsave("KM_plot.png", width = 6, height = 4, dpi = 300)

write_csv(as.data.frame(cox_model$coefficients), "cox_results.csv") write_csv(as.data.frame(summary(aft_model)$table), "aft_results.csv") write_csv(as.data.frame(rmst_result$unadjusted.result), "rmst_results.csv")

# **End of Script**

**References:**

[1] Guyot P, Ades AE, Ouwens MJ, Welton NJ. Enhanced secondary analysis of survival data: reconstructing the data from published Kaplan–Meier survival curves. BMC Med Res Methodol. 2012;12:9.
